# Interactions between Surround Suppression and Interocular Suppression in Human Vision

**DOI:** 10.1371/journal.pone.0038093

**Published:** 2012-05-25

**Authors:** Yong-Chun Cai, Shena Lu, Chao-Yi Li

**Affiliations:** 1 Department of Psychology and Behavioral Sciences, Zhejiang University, Hangzhou, China; 2 Key Laboratory for Neuroinformatics of Ministry of Education, University of Electronic Science and Technology of China, Chengdu, China; 3 Center for Life Sciences, Shanghai Institutes of Biological Sciences, Chinese Academy of Sciences, Shanghai, China; University of Regensburg, Germany

## Abstract

Several types of suppression phenomena have been observed in the visual system. For example, the ability to detect a target stimulus is often impaired when the target is embedded in a high-contrast surround. This contextual modulation, known as surround suppression, was formerly thought to occur only in the periphery. Another type of suppression phenomena is interocular suppression, in which the sensitivity to a monocular target is reduced by a superimposed mask in the opposite eye. Here, we explored how the two types of suppression operating across different spatial regions interact with one another when they simultaneously exert suppressive influences on a common target presented at the fovea. In our experiments, a circular target grating presented to the fovea of one eye was suppressed interocularly by a noise pattern of the same size in the other eye. The foveal stimuli were either shown alone or surrounded by a monocular annular grating. The orientation and eye-of-origin of the surround grating were varied. We found that the detection of the foveal target subjected to interocular suppression was severely impaired by the addition of the surround grating, indicating strong surround suppression in the fovea. In contrast, when the interocular suppression was released by superimposing a binocular fusion ring onto both the target and the dichoptic mask, the surround suppression effect was found to be dramatically decreased. In addition, the surround suppression was found to depend on the contrast of the dichoptic noise with the greatest surround suppression effect being obtained only when the noise contrast was at an intermediate level. These findings indicate that surround suppression and interocular suppression are not independent of each other, but there are strong interactions between them. Moreover, our results suggest that strong surround suppression may also occur at the fovea and not just the periphery.

## Introduction

The detectability and appearance of a target are often suppressed in the presence of a superimposed or surrounding mask. Three types of suppression have been widely reported. The first, known as surround suppression, is observed when the target is surrounded by an annular mask [1]–[5]. The strongest surround suppression can only be observed in the periphery [3], [6], [7] and is usually obtained when the target and the surround share the same properties, such as orientation and spatial frequency [1], [3]; but note that we use the term “surround suppression” to specifically refer to the surround suppression in luminance contrast, and that surround suppression for other features (such as motion) is also strong at the fovea [8], [9], [10]. The second type is overlay suppression (also called cross-orientation suppression), which is caused by a superimposed mask of any orientation presented to the same eye as the target [3], [11], [12]. The third suppressive phenomenon is referred to as interocular suppression (also called dichoptic masking), in which the target and the mask are also spatially superimposed but presented dichoptically to the two eyes [11], [13], [14]. These suppressive phenomena have also been observed in electrophysiological studies, which have demonstrated that the response of a neuron to an optimal stimulus in the classical receptive field (CRF) can be reduced by an overlapping or a flanking stimulus that alone evokes little or no response [15]–[21].

Mechanisms underlying these phenomena of suppression are not completely understood. Early studies assumed that all the three suppression phenomena derived from inhibition exerted by a pool of cortical neurons [22], but more recent reports have cast doubts on this view. Recent studies in animals [20], [21], [23], as well as in humans [11], [12], have indicated that overlay suppression is immune to contrast adaptation and can be produced with mask gratings that flicker too rapidly to elicit much of cortical response, implying that overlay suppression is generated by a subcortical mechanism. In contrast, when the mask and target stimuli are presented to different eyes, i.e., to produce interocular suppression, the suppressive effect is substantially reduced by visual adaptation and the fast-flickering masks no longer induce suppression [11], [20], [23]. This finding suggests that, unlike overlay suppression, the mechanism underlying interocular suppression arises from the cortex. For surround suppression, the issue is more complex. Recent physiological studies have shown that surround suppression can be produced by stimuli that are ineffective in driving neurons in the primary visual cortex (V1), indicating a mechanism located at a pre-cortical site [24], [25]. On the other hand, the orientation tuning and strong interocular transfer of surround suppression imply that cortical mechanism is also involved [15], [24]. All the evidence suggests that surround suppression in V1 does not originate from a single mechanism, but rather is due to a combination of thalamic and cortical mechanisms [24], [25]. In addition, psychophysical studies have also supported this multi-origin hypothesis for surround suppression [5], [26].

The majority of previous studies have concentrated on exploring the characteristics of these suppressions or evaluating the differences between them [3], [11], [20], [23], [27], while relatively little effort has been devoted to examine how different forms of suppression interact when they interfere simultaneously with a common target. Intuitively, the suppression effect of more than one type of mask should be greater than that produced by a single mask. Petrov et al (2005) showed that the addition of an orthogonal surround to the target subjected to overlay suppression results in a slight increase in the suppression effect [3] (see their Fig. 3b). Baker et al (2007), however, found that the combination of the two center suppressions (i.e., overlay suppression and interocular suppression) do not invariably result in a larger suppression effect [11]. These findings suggest that different types of suppression do not necessarily combine additively and that the ways in which they interact with one another may depend on their associated neural mechanisms. An understanding of interactions between these suppressions may help us to gain more insight into their underlying mechanisms.

How does surround suppression interact with interocular suppression? Although few studies have directly addressed this issue, related insights can be gained from studies on binocular rivalry, a phenomenon of the dynamic alternations in perception that occurs when the two eyes continuously view dissimilar images. It has been shown that the dynamics of binocular rivalry can be affected by contextual stimuli presented in proximity of rival targets [28]–[32]. In addition, Paffen et al. (2005) have reported that rivalry suppression for a speed probe is increased by the presence of a drifting surround grating, suggesting that the depth of binocular rivalry suppression can also be affected by contextual inputs [33]. Given the suggestion that binocular rivalry is closely related to interocular suppression [14], one may expect that interocular suppression may also be widely modulated by contextual information. To further explore this issue, the present study investigates how the detection of a static target is affected by a static surround when the target is concurrently subjected to interocular suppression. Previous studies have demonstrated that, for a target presented at the fovea, surround suppression does not adversely affect its detectability and only slightly impairs its apparent contrast [3], [6], [7], [27], suggesting that surround suppression is weak or even absent in the fovea. In our experiments, however, we found that the detectability of a foveal target grating that is masked by a dichoptic stimulus can be severely impaired by the addition of a surround grating. In contrast, when the interocular suppression is relieved by superimposing a common fusible feature onto both the target and the dichoptic mask [34], the surround suppression effect dramatically decreases. Our results reveal strong interactions between surround suppression and interocular suppression, implying the existence of an interplay between their underlying neural circuits. Moreover, the present results suggest that strong surround suppression, which was formerly considered to only occur in the periphery, may also appear in the fovea.

## Methods

### Observers and Ethics Statement

Six participants (P1–P6) gave written informed consent to participate in the experiments. Five of them were university students and were naïve to the purpose of the study. The other (P4) was the first author. All observers had normal or corrected-to-normal eyesight. All procedures were approved by the Research Ethics Board of University of Electronic Sciences and Technology of China.

### Apparatus and Stimuli

Stimuli were generated by using Matlab and the Psychophysics Toolbox [35], [36], and were presented on a linearized 21″ Dell UltraScan P1130 monitor (1600×1200 resolution; 85 Hz refresh rate). The grey-level resolution was 8 bits, but it was increased to 10 bits through spatial dithering using 2×2 pixel blocks. A pair of dichoptic displays was viewed through a mirror stereoscope. The effective viewing distance was 82 cm, producing a binocular field of about 10°×14°. The luminance of background screen was 35 cd/m^2^.

**Figure 1:**
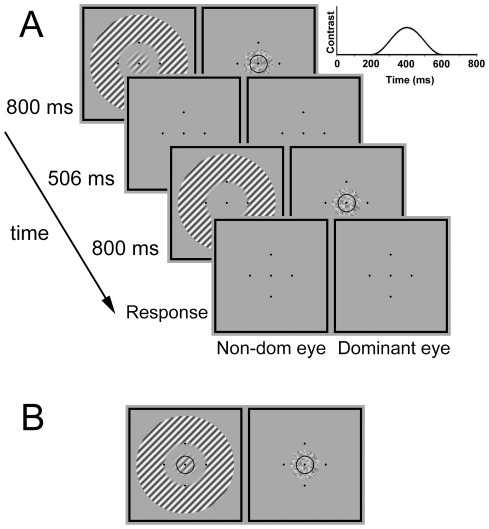
Experimental design. (A) Stimuli and sequence of events for a typical trial in Experiment 1. Observers judged which interval contained the center target grating, which was masked by a noise pattern superimposed with a black ring in the opposite eye. The target grating was either presented alone (no-surround condition) or surrounded by an annular grating (with-surround conditions). The surround and target gratings were either parallel or orthogonal to each other, and they were presented to either the same eye (monoptically) or the opposite eye (dichoptically). The figure shows the monoptic parallel condition (MP). Other conditions are not shown. The cosine curve depicts the contrast modulation of the center target in time. The five black points in each display were always presented to aid binocular alignment, and the central one served as the fixation point. (B) Typical stimuli displayed in the target interval of trials in Experiment 2. The only difference with regard to Experiment 1 was that the black ring was presented binocularly and superimposed on both the target and the noise pattern. With such a design, the ring was expected to promote summation of inputs from two eyes, thereby releasing interocular suppression.

The stimuli and experimental design are illustrated in Figure 1. The target was a circular sinusoidal grating tilted 45° clockwise from the vertical, multiplied by a raised sine function (0.49° width) with a central plateau (0.28° diameter). It therefore had an overall diameter of 1.26° and a full-width at half height of 0.77°. The dichoptic mask was a circular dynamic noise pattern superimposed with a black ring (except in Experiment 3, see below). The noise pattern had the same size as the target (but not modulated by a blurring function) and was presented to the eye opposite to the target. The noise pattern consisted of small squares of 0.07°×0.07° (4×4 pixels), each of which changed in luminance every 200 ms (17 refresh frames). The surround mask was an annular grating (1.54° inner diameter; 5.04° outer diameter) oriented either parallel (tilted 45°) or orthogonal (tilted −45°) to the target grating. It was presented either to the eye viewing the target or to the eye viewing the noise pattern, surrounding the center stimulus (i.e., the target grating or the noise pattern). Its inner border was blurred by a raised sine function of 0.25° in width. A 0.14° gap separated the center and surround stimuli. All gratings had a spatial frequency of 4.76 cycles/degree. The mean luminance of all of these stimuli was the same as that of the background.

A previous study showed that interocular suppression could be released by interocular feature matching [34]. In order to manipulate interocular suppression, a black ring (2 pixels thick; 0.77° diameter) was superimposed either monocularly only to the noise pattern (Experiment 1; Figure 1A) or binocularly to both the noise and the target grating (Experiment 2; Figure 1B). With this design, although interocular suppression was altered across the two experiments, observers had the same subjective perception.

The Michelson contrast of the surround grating was fixed at 80% in all experiments. The Michelson contrast of the noise pattern was 15% in Experiment 1 and 2. In order to explore how the strength of interocular suppression influences interactions between surround suppression and interocular suppression, the noise contrast was systematically varied from 0 to 80% in Experiment 3.

A black square frame (5.46°×5.46°; 0.09° line thick) and 5 black points (0.09°×0.09° each) were presented continuously in each display to promote stable binocular alignment. One of the points was presented in the center of each display and served as fixation point; the other four were presented on the annular grating and arranged around the center stimuli in a diamond shape, each placed 1.02° away from the center fixation point (Figure 1).

### Procedure

The target grating and the dichoptic mask were presented to the fovea of the non-dominant and dominant eyes, respectively. The annular grating was presented either to the same eye as the target (monoptic conditions) or to the opposite eye (dichoptic conditions), surrounding the center stimuli. We used a two-interval forced-choice (2IFC) procedure, in which the two test intervals each lasted for 800 ms and were separated by a 506 ms blank interval (Figure 1A). Both the surround mask (except for the no-surround condition) and the center dichoptic mask were presented throughout the two entire test intervals. The target grating was randomly presented in one of the test intervals. To avoid any abrupt onset/offset effect, the target grating appeared 200 ms later than the mask stimuli and ramped on and off with a 400 ms cosine temporal window (the curve in Figure 1A illustrates the temporal modulation of the target contrast). Each test interval was signaled by a beep. Observers were asked to maintain fixation throughout the entire trial and to judge which interval contained the target. Audio feedback was given in the event of an incorrect response.

Target detection thresholds corresponding to the correct rate of 75% were determined using a QUEST staircase procedure [37], [38]. In Experiments 1 and 2, all conditions with respect to eye-of-origin and orientation of the surround grating were randomly interleaved. In Experiment 3, trials were blocked by the surround conditions and noise contrasts. For each observer, each threshold measurement was estimated from a staircase of 50 trials and each data point was averaged from at least four repeated measurements.

## Results

### Experiment 1: surround suppression when accompanied by interocular suppression

In this experiment, we explored how the detection of the target grating was affected by surround gratings when the target was undergoing interocular suppression. The center target grating, which was concurrently masked by a dichoptic stimulus (i.e., a noise pattern with a ring superimposed on it; Figure 1A), was either presented alone (no-surround, NS) or surrounded by a monoptic parallel grating (MP), a monoptic orthogonal grating (MO), a dichoptic parallel grating (DP), or a dichoptic orthogonal grating (DO). The surround effects could be revealed by comparing the target detection thresholds of with-surround conditions (i.e., the MP, MO, DP and DO conditions) with that of the no-surround condition (i.e., the NS condition).

**Figure 2:**
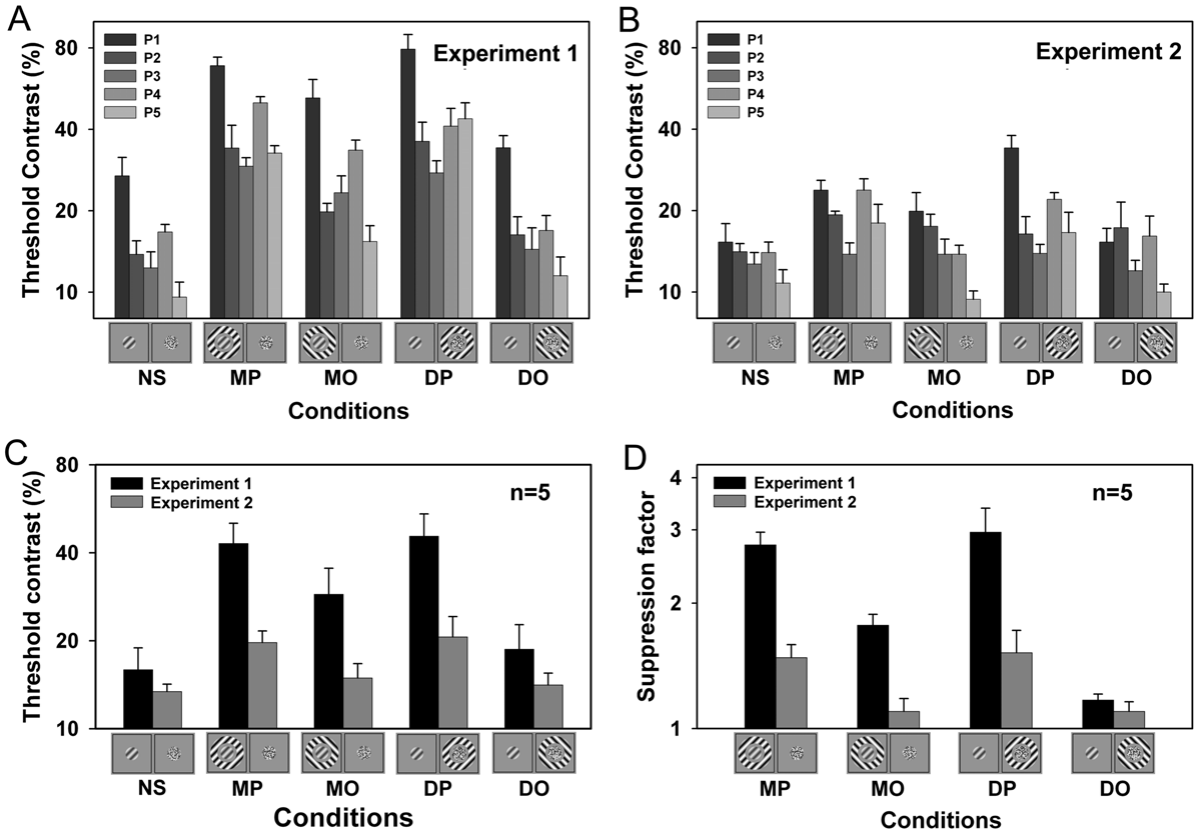
Results of Experiments 1 and 2. Individual detection thresholds of the target grating for each surround condition are shown for (A) Experiment 1 and (B) Experiment 2. Averaged results (n=5) for the two experiments are shown in panel (C). Horizontal axis represents different surround conditions: NS, no surround; MP, monoptic parallel surround; MO, monoptic orthogonal surround; DP, dichoptic parallel surround; and DO, dichoptic orthogonal surround. Suppression factors, defined as the ratio of with-surround to no-surround thresholds, for each with-surround condition of Experiments 1 and 2 are shown in panel (D). An iconic depiction of the stimuli for each condition is illustrated below the horizontal axis (note that the black ring is not shown in iconic depictions). Error bars represent standard error of the mean.

Figure 2A shows detection thresholds for the five conditions for individual observers, and Figure 2C (black bars) shows the averaged results. An one-way repeated ANOVA revealed a significant difference among experimental conditions (*F*(4, 16)=21.1, *p*<0.001). Post hoc comparisons with the Fisher's least significant difference (LSD) test showed that detection thresholds under all with-surround conditions, except the DO condition, were significantly higher than that under the no-surround condition (MP: *p*<0.005; MO: *p*<0.01; DP: *p*<0.03; DO: *p*>0.07), meaning that surround suppression was produced under these conditions.

To evaluate the strength of surround suppression, a suppression factor, defined as the ratio of with-surround to no-surround thresholds, was calculated for each with-surround condition (see Figure 2D, black bars). The suppression factor would have a value greater than 1 if there was surround suppression. A two-way repeated ANOVA was conducted on the suppression factor with eye-of-origin (monoptic vs. dichoptic) and orientation (parallel vs. orthogonal) of the surround grating as factors. This analysis revealed a significant main effect of orientation (*F*(1,4)=20.2, *p*<0.02) but no effect of eye-of-origin (*F*(1,4)=1.1, *p*>0.35) and a significant interaction between the two variables (*F*(1,4)=13.9, *p*<0.02). A post hoc Fisher's LSD test showed that the parallel surround always produced strong suppression, irrespective of the eye to which it was presented (i.e., there was no difference between the MP and DP conditions, *p*>0.50). However, the orthogonal surround could produce significant suppression only when it was presented to the same eye as the target grating (i.e., the MO condition). This indicates that the suppression from a parallel surround can transfer across the eyes, but the suppression from an orthogonal surround can not. This finding suggests that the parallel suppression originates from the site(s) beyond the binocular convergence, while the orthogonal suppression occurs at a monocular stage, which is consistent with a previous suggestion that the two kinds of surround suppression have distinct sources [5], [24].

In this experiment, we obtained strong suppression effects (except for the DO condition), especially for parallel surrounds (i.e., the MP and DP conditions) by which detection thresholds were elevated by nearly a factor of 3. This is in contrast to earlier studies demonstrating that surround stimuli only slightly impair the perceived contrast of a foveal target [5], [39] and have no effect on the detection of a foveal target [3], [6], [7], [27]. The question therefore arises of how the surround suppression, which was believed to be weak or even absent in the fovea, could be so effective in preventing the center target from breaking the suppression of dichoptic stimuli. We assume that the interocular suppression produced by the center dichoptic mask might interact with the suppression produced by the surround gratings and that this interaction might make the otherwise weak surround suppression appear strong.

There is another possibility, however, that the large surround suppression effects observed here are not due to interactions between surround suppression and interocular suppression, but simply due to the specific stimulus configurations we used. For example, the detection of a target masked with a noise might be more prone to be influenced by contextual inputs, thereby allowing us to obtain stronger surround suppression. The effective contrast of the target might be reduced by the dichoptic mask to low levels at which surround suppression has been reported to be stronger [2], [6]. It is therefore possible that the strong surround suppression effect would be observed as long as the target was interfered with by a similar masking stimulus, even if the interocular suppression was weak or absent. The next experiment was designed to test this possibility.

### Experiment 2: surround suppression decreased when interocular suppression was released

In this experiment, we aimed to examine the effects of surround gratings when interocular suppression was abolished or weakened. Meese and Hess (2005) have reported that the suppression induced by a binocular mask is much weaker than that induced by a dichoptic mask [34]. This finding indicates that interocular suppression is released when the dichoptic mask is fully fused with the same mask in the other eye. Studies involving binocular rivalry have also shown that rivalry is reduced by the introduction of fusible contours to the conflicting images in the two eyes [40], [41]. This result suggests that even partially fusible features can promote the summation of the dissimilar images in the two eyes and thus lead to the reduction of the interocular competition. Given the close correlation between interocular suppression and binocular rivalry [14], it is plausible to expect that interocular suppression can also be relieved by partially fusible features.

In this experiment, a black ring was superimposed on the target grating to match the same ring on the dichoptic noise pattern (Figure 1B). With this manipulation, the interocular suppression was expected to be attenuated. Other aspects and observers' subjective perception were the same as those in Experiment 1. If the strong surround suppression observed in Experiment 1 was not a result of an involvement of interocular suppression, but due to the specific stimulus configurations, the large suppressive effects would also be observed in this experiment.

Figure 2B shows detection thresholds for individual observers, and Figure 2C (gray bars) shows the averaged thresholds. An one-way repeated ANOVA revealed a significant difference among various conditions (*F*(4, 16)=4.9, *p*<0.01). A post hoc analysis (Fisher's LSD test) showed that, compared with the no surround condition, the target detection threshold in the MP condition was significantly elevated (*p*<0.02), whereas other with-surround conditions had no significant effect (all *p*s>0.08). A two-way ANOVA analysis revealed that the suppression factors in this experiment (Figure 2D, gray bars) were significantly smaller than those in Experiment 1 (Figure 2D, black bars; *F*(1, 4)=36.9, *p*<0.005). In particular, for parallel surround conditions, the introduction of the binocular matching ring led to a remarkable decrease in the surround suppressive effect from a factor of about 3 to below 1.5 (MP: *p*<0.001; DP: *p*<0.05; post hoc Fisher's LSD test). These results demonstrate that the manipulation of interocular suppression can greatly influence the effect of surround suppression. Even though this experiment had very similar stimulus configurations to those in Experiment 1, surround suppression was substantially decreased by releasing the interocular suppression. This suggests that the robust surround suppression effects found in Experiment 1 can not be attributed to the specific surround and center stimuli, but are mainly due to the involvement of interocular suppression.

### Experiment 3: varying the contrast of dichoptic noise

Taken together, Experiments 1 and 2 indicate that surround suppression and interocular suppression are not two independent suppression phenomena, but they interact with one another. In this experiment, we further investigated whether these interactions depend on the strength of interocular suppression. To address this question, we examined how surround suppression varied with increasing strength of interocular suppression, which was achieved by systematically increasing the contrast of dichoptic noise. Six noise contrast levels (0%, 5%, 10%, 20%, 40%, and 80%) were tested. Other aspects were the same as in Experiment 1, except that no black ring was presented.

**Figure 3:**
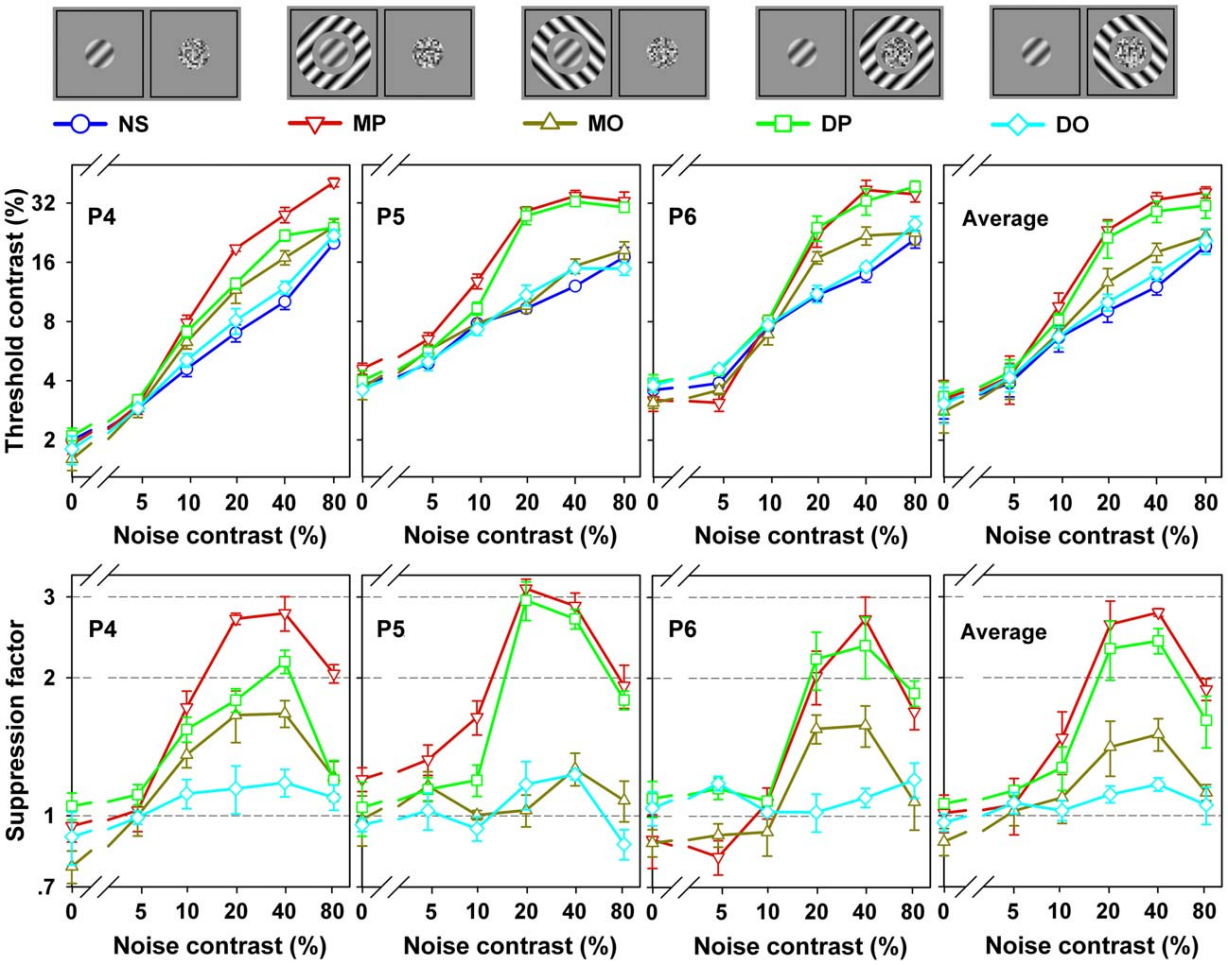
Results of Experiment 3. Detection thresholds (upper panels) and suppression factors (lower panels) are plotted as functions of noise contrast for each surround condition. Results for three participants are arranged in columns; the last column shows the average result. An iconic depiction of the stimuli for each condition and the corresponding symbol are shown above the plots. Error bars represent standard error of the mean.

The individual and averaged results for the three participants are shown in Figure 3, in which target detection thresholds (upper panels) and suppression factors (lower panels) are plotted as a function of noise contrast, respectively. As expected, the detection threshold in all conditions increased with noise contrast. The surround effects changed with varying levels of the noise contrast and the variation in surround suppression depended on surround configuration (MP: *F*(5,10)=43.4, *p*<0.001; MO: *F*(5,10)=4.5, *p*<0.03; DP: *F*(5,10)=11.8, *p*<0.001; DO: *F*(5,10)=1.3, *p* > 0.3; One-way ANOVA with repeated measures). When the noise contrast was zero (i.e., no interocular suppression), no surround suppression was observed for each surround configuration, consistent with a previous suggestion that surround suppression is absent in the fovea [3], [6]. As noise contrast increased, for the parallel surround conditions, surround suppression initially increased and reached a maximum at an intermediate contrast level (20%–40%), after which it dropped at higher noise contrasts (Figure 3, lower panels; downward triangles for MP, squares for DP). For the orthogonal surround conditions, however, surround suppression was weak and did not dramatically change with an increase in noise contrast (Figure 3, lower panels; upward triangles for MO, diamonds for DO). The dependence of surround suppression (at least for iso-orientation surround suppression) on dichoptic noise contrast again indicates that surround suppression and interocular suppression are not independent, but interact, and interactions between them are strongest only when the interocular suppression is modest.

## Discussion

Previous studies reported that surround suppression is strong only in the periphery, but weak or even absent in the fovea. In the study presented here, we tested the effect of a surround mask on the detection of a foveal target that was simultaneously interfered with by an interocular mask. We observed strong surround suppression, especially in parallel surround conditions under which the target detection threshold could be elevated by about a factor of 3. However, when interocular suppression was released by presenting a fusion ring to the two eyes, the strong surround suppression effect was no longer observed, suggesting that the interocular suppression plays a critical role in producing the robust surround suppression. In another experiment, we further found that the strength of surround suppression depends on the contrast of the interocular mask. The strongest surround suppression was found only when the dichoptic mask was at a medium contrast level. These findings suggest that surround suppression and interocular suppression are not two independent suppression phenomena, but that there exist interactions between them.

### Possible explanations for the present findings

How can we explain the present finding that the otherwise weak foveal surround suppression is enhanced to a high level when it is accompanied by interocular suppression? Because no neurophysiological study, to our knowledge, has ever investigated the two forms of suppression in a single experiment, the neural mechanisms underlying their interactions remain unknown. At this point, we can only offer some speculations.

Both interocular suppression and orientation-specific surround suppression have been proposed to originate from the visual cortex and to be generated via inhibitory GABAergic interneurons [23], [42]. It is possible that a target neuron may receive inhibitory projections from both sources of the two types of suppression. Because of the complex processing in the visual cortex [43], when the target neuron concurrently receives the inhibitory inputs from the two suppressions, the suppressive effects may be combined nonlinearly, such that the otherwise weak surround suppression is amplified to a strong level. The nonlinearity of the combination might depend on the strength of the two suppressions. Thus, the alteration of interocular suppression would result in the variation of surround suppression.

Alternatively, our result may be attributed to high-level cognitive processes. A psychophysical study has shown that surround suppression is dramatically affected by attention: compared with the full attention condition, the suppression effect in the poor attention condition is increased by about a factor of 4 [44]. Likewise, animal studies have revealed that contextual modulations of neurons from striate as well as extrastriate visual areas are modulated by visual attention [45]–[47]. Previous studies that failed to observe strong surround suppression in the fovea required subjects to fully concentrate their attention on the foveal target. This heightened attention might reduce surround suppression to a low level. In the present study, however, the foveal target was suppressed from visual awareness by the dichoptic mask: observers could not focus their attention on the target grating, but instead on the dichoptic mask. Therefore, the attentional modulation was eliminated and, in turn, the surround suppression was enhanced relative to previous results. Although this explanation sounds plausible, it cannot, however, account for the decrement in the surround suppression at the high contrast of a dichoptic mask (see Figure 3).

### Source of surround suppression and effects on overlay suppression

At which stage in the visual pathway do the surround stimuli exert their influence on interocular suppression? A previous study suggested that interocular suppression originates from a stage prior to complete binocular combination [11]. It is reasonable to expect that surround modulation on interocular suppression also takes place at the monocular stage where interocular suppression arises. Our present results also support this suggestion. Neurons located at a site beyond binocular summation should be unable to distinguish between the center stimuli of Experiments 1 and 2. Hence, if the surround stimuli exerted their effects at a binocular stage, we should have obtained the same contextual effects in both of the experiments. However, the surround suppression effects obtained from the two experiments were markedly different, suggesting surround suppression occurs before binocular summation.

**Figure 4:**
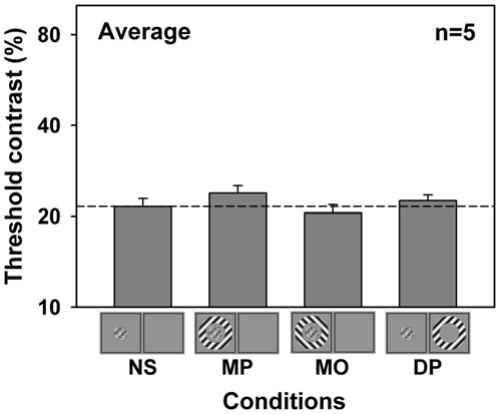
Surround effects on overlay suppression. This preliminary experiment was performed to examine how surround suppression exerts effects on overlay suppression. In this experiment, the center target was blended into the center noise. That is, the target and noise were presented in the same eye and overlay suppression was expected to occur. All other factors were similar to Experiment 1 (but note that the dichoptic orthogonal surround [DO] condition was not tested). Averaged result of 5 observers is depicted. The dashed line indicates no surround suppression. An iconic depiction of the stimuli for each condition is illustrated below the horizontal axis. Error bars represent standard error of the mean. The difference among experimental conditions was significant (*F*(3,12)=6.6, *p*<0.01, one way ANOVA with repeated measures). However, post hoc comparisons showed that only the threshold in the monocular parallel surround condition (MP) was significantly elevated by a factor of 1.1 relative to the no-surround condition (NS) (MP: *p*<0.02; MO: *p*>0.1; DP: *p*>0.2; Fisher's LSD test). This is in contrast to the strong surround suppression effects on interocular suppression found in Experiment 1.

Furthermore, in a preliminary experiment, we found that when the noise mask was superimposed onto the target grating in the same eye (overlay suppression), the effects of surround suppression on target detection were very limited (see Figure 4). This is in contrast to the powerful surround suppression found in Experiment 1, in which the mask and target were presented to opposite eyes. This result suggests that when the target has been combined with the mask, it hardly receives suppression from the surround. Therefore, the strong surround suppression observed in our main experiments can only be elicited at the stage before the dichoptically presented target and mask being combined together. Moreover, this result indicates that overlay suppression and interocular suppression are affected differently by the surround suppressive input, suggesting that they are based on different mechanisms [11], [20], [23].

We have argued above that surround suppression exerts its effects on interocular suppression prior to binocular combination. The question then can be raised of the stage at which the surround suppressive signal originates. In the present study, the most robust suppressive effects were caused by the parallel surround grating, regardless of whether it is presented to the same eye as the target or to the other eye, i.e., the iso-orientation surround suppression can transfer across the eyes. This strong interocular transfer of surround suppression has also been reported in previous psychophysical [4], [5], [48] (but see [49]) and physiological studies [15]. These findings suggest that the source of iso-orientation surround suppression is located at a binocular stage. Given the above argument that surround suppressive influence is implemented prior to binocular combination, we speculate that iso-orientation surround suppression might be generated through inter-area feedback projections from higher visual areas (such as the extrastriate cortex) or interlaminar feedback from layers containing binocular neurons within V1.

### Interocular suppression and binocular rivalry

In the present study, the center target and the dichoptic mask were briefly presented, and the detectability of the target was impaired by the mask because of interocular suppression. However, if the dissimilar stimuli were continuously presented to different eyes, we would then observe another suppression phenomenon – binocular rivalry, in which perception alternates between the images of the two eyes every few seconds.

Because binocular rivalry and interocular suppression are produced under very similar stimulus condition, they are usually thought to involve common neural mechanisms. One piece of evidence has been provided by Baker and Graf. They reported that the mean dominance durations in binocular rivalry correlate with the magnitude of interocular suppression within, as well as between, observers. [14].

Here, we found that interocular suppression was influenced by surround inputs, especially when the surround and the target had the same orientation. If binocular rivalry shares common mechanisms with interocular suppression, it should also be greatly affected by contextual stimuli. In line with this prediction, Paffen et al. (2005) have reported that the discrimination of a speed probe, which was presented on a drifting grating undergoing binocular rivalry suppression, is further impaired when a surround motion is added, suggesting that binocular rivalry suppression can be deepened by contextual stimuli [33]. The effect of the moving surround is strongest when the surround shares the same motion direction as the rival grating [33]. This is very similar to the present finding that the surround effect on interocular suppression is strongest when surround grating and the center grating share the same orientation.

Furthermore, the dynamics of binocular rivalry has also been found to be influenced by contextual stimuli. For example, the predominance of a rival stimulus is greatly decreased by the presence of a context sharing the same properties (such as orientation, motion, and color) as the rival target [28]–[32]. These contextual influences are believed to be mediated by surround suppression that inhibits the neural activity of the rival target and, in turn, reduces its predominance during rivalry [29], [31]. In addition, the decrease in target predominance is achieved by prolonging its mean rivalry suppression duration while leaving mean dominance duration unaffected [30], suggesting that surround suppression can exert its effects only when the target is suppressed from awareness by its competitor. This resembles the present finding that surround suppression is strong only when it is accompanied by interocular suppression, but largely reduced when a binocular ring prompts the fusion of the target with the dichoptic mask (Experiment 2). These similarities between binocular rivalry and interocular suppression support the hypothesis that they arise from common mechanisms.

### Conclusions

We have shown that the detection of a foveal target that is simultaneously undergoing interocular suppression is further strongly impaired by the presence of surround stimuli. This surround suppression effect is influenced by the manipulation of the interocular suppression and varies with the strength of interocular suppression. These results suggest that surround suppression and interocular suppression are not independent of one another, but that there are interactions between their underlying neural mechanisms. In addition, our results also suggest that surround suppression does not only prevail in the periphery, but can, under specific conditions, also exhibit a strong effect in the fovea.
